# Increased Susceptibility to Dextran Sulfate Sodium Induced Colitis in the T Cell Protein Tyrosine Phosphatase Heterozygous Mouse

**DOI:** 10.1371/journal.pone.0008868

**Published:** 2010-01-25

**Authors:** Syed-Wajahat Hassan, Karen M. Doody, Serge Hardy, Noriko Uetani, Denis Cournoyer, Michel L. Tremblay

**Affiliations:** 1 Rosalind and Morris Goodman Cancer Centre, McGill University, Montreal, Canada; 2 Department of Medicine, Division of Experimental Medicine, McGill University, Montreal, Canada; 3 Department of Biochemistry, McGill University, Montreal, Canada; 4 Department of Oncology, McGill University, Montreal, Canada; New York University, United States of America

## Abstract

T cell protein tyrosine phosphatase (TC-PTP / PTPN2) is an enzyme that is essential for the proper functioning of the immune system and that participates in the control of cell proliferation, and inflammation. We previously observed that TC-PTP^−/−^ mice display various immunodeficiencies, hypersensitivity to LPS and die within three weeks of birth due to anemia and widespread inflammation. A recent analysis of the Wellcome Trust Case Control Consortium (WTCC) genome wide scan data, reported in 2007, indicated a potential role for TC-PTP in inflammatory bowel disease (IBD). To further investigate the potential role of TC-PTP in IBD, we studied heterozygous TC-PTP mutant mice challenged with dextran sulfate sodium (DSS) in their drinking water. In comparison to control animals, we observed significant changes in the colon mucosa of DSS-treated TC-PTP^+/−^ mice, in the ratio of colon to body weight, as well as an up-regulation of mRNA transcripts for IL-6, IL-23, 1L-12β, IFN-γ, TNF-α. Moreover, up-regulation of serum IL-6 levels in DSS-treated TC-PTP^+/−^ mice confirms that mice with a single copy of the TC-PTP gene display increased susceptibility to systemic inflammation due to bowel epithelial erosion resulting from DSS challenge. Our findings support the lack of modulation of Janus kinases 1 and 3 (Jak1, Jak3), and the downstream signal transducer and activator of transcription 1,3 and 5 (Stat1, Stat3, Stat 5) by PTPN2 in the development of IBD like condition. Pathological and molecular analysis reveal that the deficiency of TC-PTP results in pro-inflammatory condition in the bowel of heterozygous TC-PTP^+/−^ mice. These novel findings in TC-PTP hemi-deficiency support the hypothesis that TC-PTP is an important regulator of inflammatory cytokine signaling and that it may be implicated in the pathophysiology of IBD.

## Introduction

Ulcerative colitis and Crohn's disease are the main two types of inflammatory bowel disease (IBD). Colitis is a well established risk factor for the development of colorectal cancer, the 3rd leading cause of cancer in North America in both men and women with respect to both the incidence rate and estimated death rates [1], [2]. IBD is associated with both colitis and ileitis. [1], [3]–[7]. The cause(s) of colitis in IBD remains unknown, although many theories have been proposed, and links with genetic loci have been postulated. Individuals with IBD tend to have abnormalities of the immune system. More than 1 million Americans and more than 2 millions Europeans suffer from IBD [1]. Crohn's Disease, the 3^rd^ ranking disease in a genome-wide association study performed by the Wellcome Trust Case Control Consortium (WTCC) in 2007 [2], was found associated with a SNP in a region of chromosome 18p11 with the only gene lying in this region being *PTPN2*, the encoding locus for the T-cell protein tyrosine phosphatase (TC-PTP) [2]. Moreover, other recent genetic studies have also shown links between IBD and the TC-PTP (*PTPN2*) locus [1]–[7].

TC-PTP is a classical non-receptor protein tyrosine phosphatase that can regulate pro-inflammatory cytokine signaling [8]. *PTPN2*-knockout mice have altered hematopoiesis affecting several lineages [9] and develop severe inflammation within two weeks of birth [8]–[11]. TC-PTP was one of the first members of the PTP gene family identified. Although originally cloned from a T cell cDNA library, it is ubiquitously expressed at all stages of mammalian development and in most embryonic and adult tissues [12]. The mouse isoform of TC-PTP was later cloned in 1992 [13]. *PTPN2* is located on chromosomal region 18p11.2–11.3 in humans and in a syntenic region in mice [14]. The 45 kDa form of TC-PTP (45 kDa) contains a nuclear localization signal N-terminal to its catalytic domain, and is encoded by the main detectable splice form in mice [13].

The systemic inflammation in TC-PTP^−/−^ mice and various genetic studies focusing on the association of the *PTPN2* locus with IBD led to the hypothesis that the absence of the enzymatic activity or decreased expression of TC-PTP may play a role in many chronic inflammatory conditions. We evaluated the role of TC-PTP as a modulator of inflammation in IBD relevant conditions *in vivo*, using a dextran sulfate sodium (DSS)-induced colitis mouse model. DSS causes direct damage to the epithelial barrier of the colon leading to cellular dysfunction, impaired epithelial repair and altered epithelial barrier [15], [16]. We compared TC-PTP ^+/−^ and wild-type mice treated with dextran sulfate sodium verses non-treated controls [15], [16]. We observed that TC-PTP ^+/−^ mice were more sensitive to DSS treatment than TC-PTP^+/+^ mice as shown by histopathological alterations of large bowel, increased cytokine expression in serum and cytokine signaling in the large bowel. Heterozygosity of TC-PTP may thus prime the immune system to respond more readily to irritants such as DSS resulting in a more severe inflammatory condition. This may partially explain the association of TC-PTP with chronic inflammatory diseases such as IBD [1]–[7]. TC-PTP is a key modulator in inflammation, and by detecting or controlling its expression or downstream events we may open new avenues in the diagnosis and treatments of inflammatory bowel diseases like colitis.

## Results

### Increased Weight Loss and Decreased Colon Weight of TC-PTP ^+/−^ Mice during DSS-Induced Colitis

To evaluate the role of TC-PTP in bowel inflammation, we treated wild type (WT) and heterozygous TC-PTP ^+/−^ mice with DSS to induce colitis. We compared four groups of animals: DSS-treated TC-PTP ^+/−^ and TC-PTP^+/+^ mice, and non-treated TC-PTP ^+/−^ and TC-PTP^+/+^ mice. TC-PTP^−/−^ mice were not subjected to DSS-treatment as they die shortly after weaning [8]–[11] and cannot live long enough to complete the course of DSS treatment. However, untreated TC-PTP^−/−^ mice were used as controls in some experiments.

**Figure 1:**
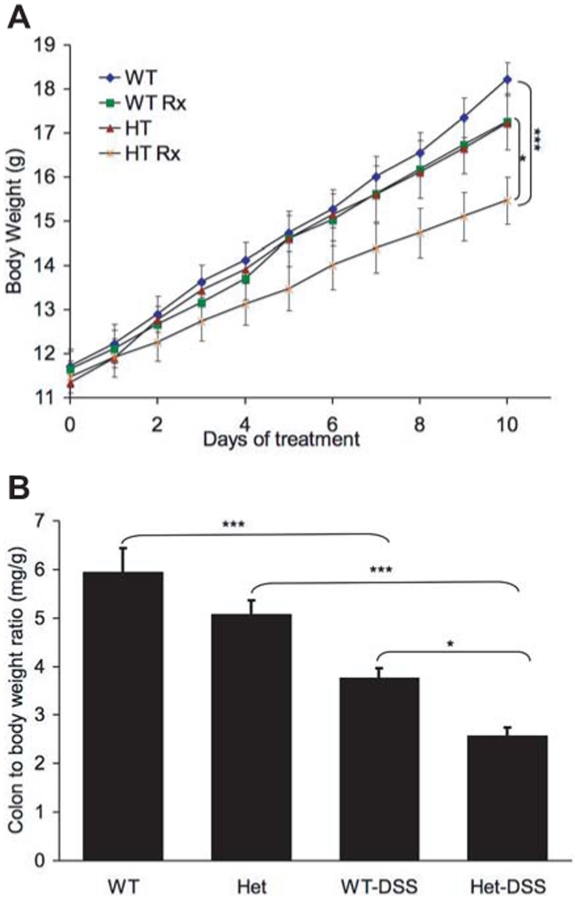
Increased response to DSS treatment in the presence of only one allele of TC-PTP. **A**. Increased weight loss of TC-PTP ^+/−^ mice during DSS-induced colitis. At 19 days of age mouse body weight was 11–12 g followed by DSS treatment. Body weight of DSS-treated TC-PTP ^+/−^ (Het-DSS) (2.58±0.4) mice were significantly lower than, TC-PTP^+/−^ (Het) (5.0±0.8) and TC-PTP^+/+^ (WT-DSS) (3.78±0.5) mice ***p<0.0002. DSS-treated TC-PTP^+/−^ mice compared to DSS-treated TC-PTP^+/+^ mice *p<0.001. Data represent the mean of three groups of three animals each (N = 9). **B.** Ratio of colon weight of DSS-treated TC-PTP ^+/−^ (Het-DSS) mice were significantly lower than DSS-treated TC-PTP^+/+^ (WT-DSS) mice *p*<0.05. In the case of TC-PTP^+/−^ (Het) vs. DSS-treated TC-PTP^+/−^ (WT-DSS) mice p<0.001, and *p*<0.001 in comparison of TC-PTP^+/+^ vs. DSS-treated TC-PTP^+/+^ mice.

We observed a significant decrease in the body weight of DSS-treated TC-PTP^+/−^ mice compared to DSS-treated TC-PTP^+/+^ mice (*p<0.001). In addition there was a decrease in body weight in DSS-treated TC-PTP ^+/−^ mice, throughout the course of DSS treatment, in comparison to non-treated TC-PTP ^+/−^ (*p<0.001) and TC-PTP^+/+^ (***p<0.0002) (Figure 1A). Furthermore the ratio of colon to body weight of DSS-treated TC-PTP^+/−^ mice was significantly lower than those of DSS-treated TC-PTP^+/+^ mice (* *p*<0.05) (Figure 1B). Similarly, a significant decrease in colon to body weight ratio was observed in DSS-treated TC-PTP^+/−^ mice in comparison to non-treated TC-PTP^+/−^ mice (*** *p*<0.001), as well as in DSS-treated TC-PTP^+/+^ compared to non-treated TC-PTP^+/+^ mice (*** *p*<0.001).

### DSS-Treated TC-PTP^+/−^ Colons Display Histological Alterations and Elevation of Peripheral Blood Lymphocytes and Neutrophils

**Figure 2:**
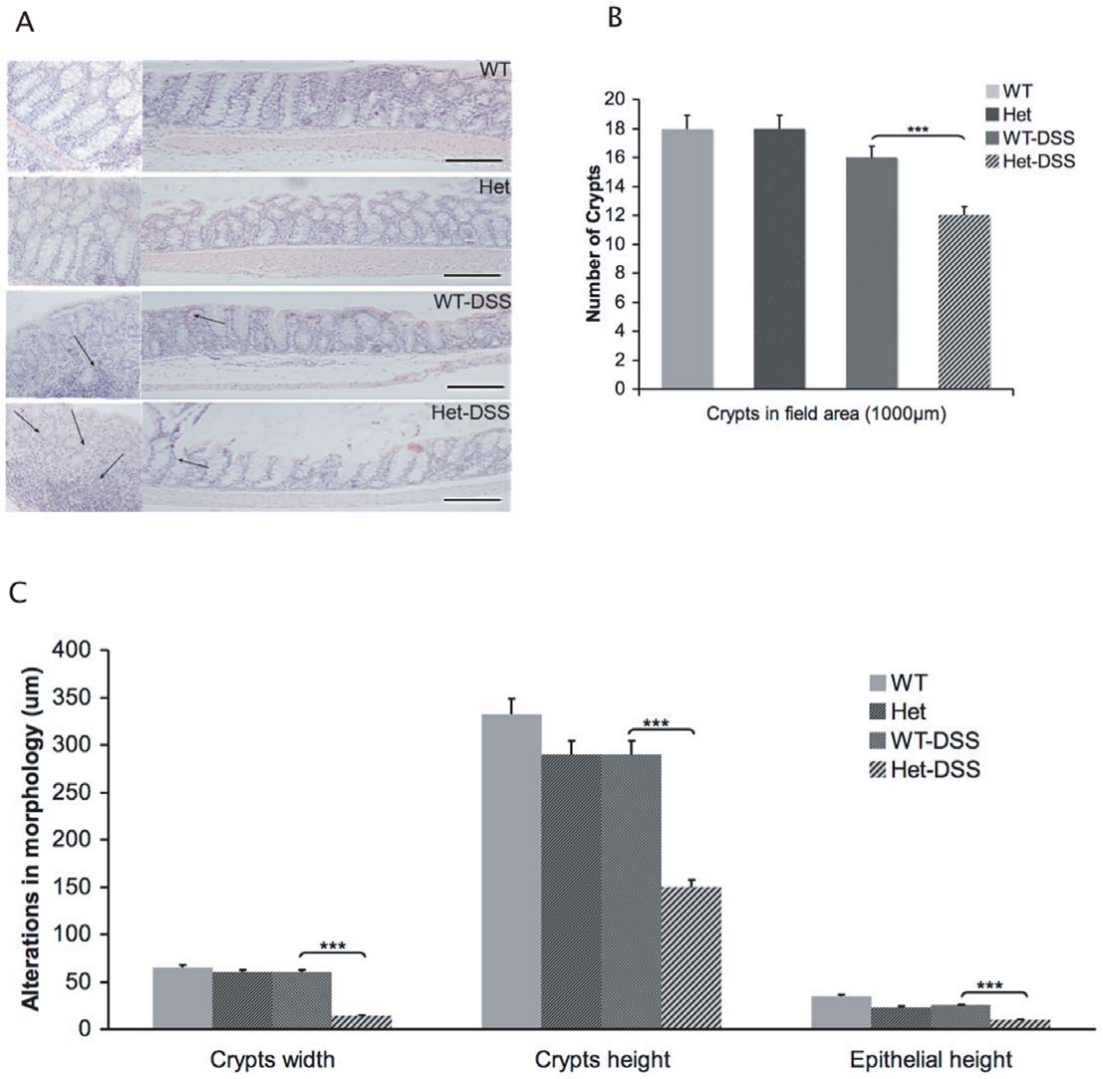
Loss of colorectal crypt-villi. **A.** Histopathological features of the mouse colon in association with colitis. Bar = 1000 µm. From top to bottom: TC-PTP^+/+^(WT), TC-PTP^+/−^ (Het), DSS-treated TC-PTP^+/+^(WT-DSS) and DSS-treated TC-PTP^+/−^ (Het-DSS). In TC-PTP^+/+^(WT) crypts are straight and the base of the glands reach the muscularis mucosae. Crypt-villus axes are normal and epithelium layer is intact. In DSS-treated TC-PTP^+/−^ (Het-DSS) mice ingestion of DSS caused crypt destruction, Numerous neutrophils were present in the section (arrows). Loss of the surface epithelial layer is obvious, while changes in crypt-villus axis and colon tissue architecture are observed. **B.** The number of crypts per field, field is 1000 µm in colorectal section of bowel. The number of crypts in DSS-treated TC-PTP^+/−^ (Het-DSS) mice were significantly lower than DSS-treated TC-PTP^+/+^ (WT-DSS) mice *p*<0.002. **C.** Crypts in field, crypts width, crypts height and epithelial height were quantified. Data represent mean and SD of three groups of three animal each (N = 9).

**Figure 3:**
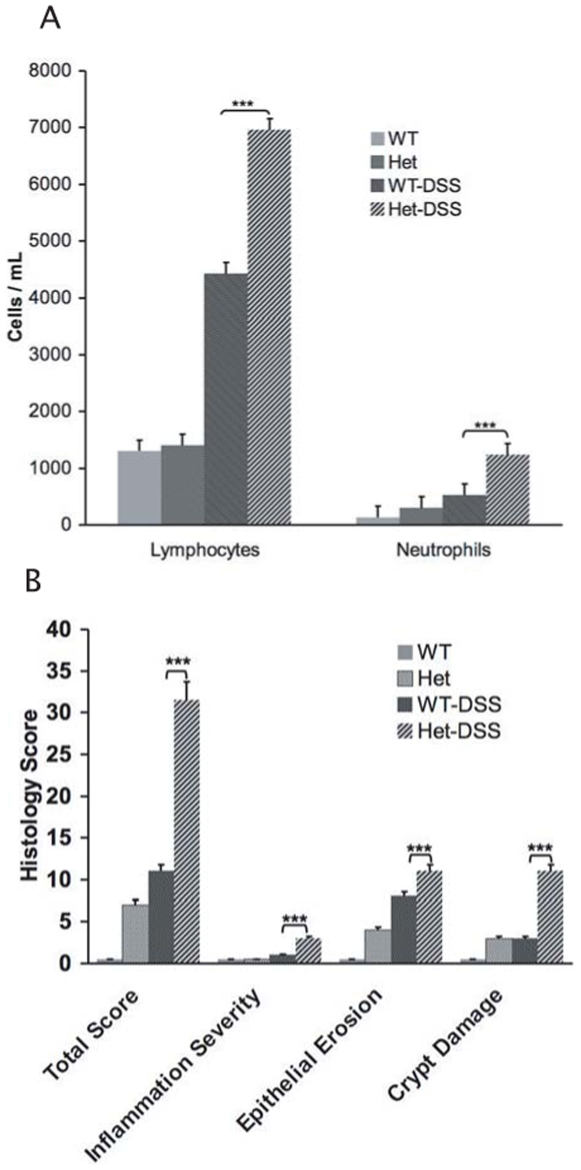
Blood cell counts and histological analysis of colons. **A.** Increase in numbers of lymphocytes *p*<0.0114 and neutrophils *p*<0.0001 in DSS-treated TC-PTP ^+/−^ mice compared to DSS-treated TC-PTP^+/+^. **B.** The mean histology activity scores of colon tissue sections of TC-PTP^+/+^(WT) (0.3±0.34), TC-PTP^+/−^ (Het) (7±0.55), DSS-treated TC-PTP^+/+^(WT-DSS) (12±0.48) and DSS-treated TC-PTP^+/−^(Het-DSS) (32±0.67) mice. Analysis was performed blinded by pathologist on a scale from 0 to 40 (see Methods).

To further investigate the phenotypes seen in DSS-treated TC-PTP heterozygous mice, we evaluated the degree of inflammatory activity by histo-pathological examination of the colons from animals with the different genotypes. Colon tissue sections on WT controls demonstrated normal crypt parallelism and intact surface epithelium (Figure 2A, B and C). Tissues collected from DSS-treated mice demonstrated crypt distortion and neutrophilic infiltration (Figure 2A, B and C). Alterations in morphology during colonic injury in DSS-treated TC-PTP^+/−^ mice compared to DSS-treated TC-PTP^+/+^, non-treated TC-PTP^+/−^ and non-treated TC-PTP^+/+^ mice showed significant increase in epithelial erosion and crypt damage, as shown in (Figure 2C). Colons from DSS-treated TC-PTP^+/−^ mice also showed significantly more pronounced decrease in number of crypts (*p*<0.001), crypt width (*p*<0.003), crypt height (*p*<0.005) and epithelial height (*p*<0.0002) as compared to DSS-treated TC-PTP^+/+^, as shown in Figure 2C. Histo-pathological scoring for assessment of inflammatory changes was 32 out of a maximum of 40 in DSS-treated TC-PTP ^+/−^ mice, compared to 12 in DSS-treated TC-PTP^+/+^, (*p*<0.0003), as shown in Figure 3B. Likewise, differential peripheral white blood cell counts revealed a significant increase in lymphocyte (*p*<0.0114) and neutrophils (*p*<0.0001) counts in DSS-treated TC-PTP ^+/−^ mice compared to DSS-treated TC-PTP^+/+^ (Figure 3A).

### Hyperactivation of STAT and JAKs in TC-PTP^+/−^ Colon upon DSS-Treatment

**Figure 4:**
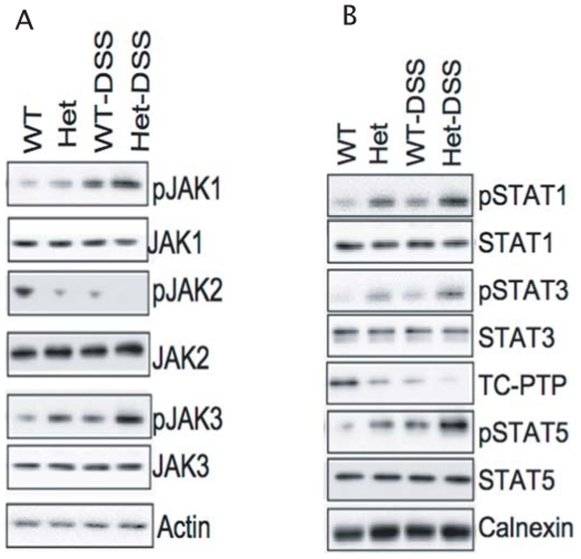
Jak/Stat signaling pathways. Immunoblotting was used to observe total protein levels and protein phosphorylation levels of JAK1, JAK3, STAT1, STAT3 and STAT5.

Key signaling molecules involved in the inflammatory process includes the janus kinases (Jaks) and the downstream effectors signal transducers and activators of transcription (STATs). Since TC-PTP has been shown to be a major regulator of this pathway, and given that DSS-induced colitis is known to trigger inflammation in the bowel and activate these pathways [8]–[11], we examined the phosphorylation status of various Jaks and STATs in colonic cells from mice of various TC-PTP genotypes challenged with DSS treatment. Hence, bowel sections were isolated for immunoblotting. DSS treatment in TC-PTP^+/−^ mice results in hyper-phosphorylation of JAK1, JAK3, STAT1, STAT3 and STAT5 while there was a decrease in JAK2 activity (Figure 4). Interestingly, both TC-PTP^+/+^ and TC-PTP^+/−^ mice showed a decrease in protein levels of TC-PTP in the bowel following DSS treatment with levels being at the limit of detection by Western blotting in treated TC-PTP^+/−^ mice.

### Up-Regulation of Pro-Inflammatory Cytokines in the Colon of DSS-Treated TC-PTP ^+/−^ Mice

**Figure 5:**
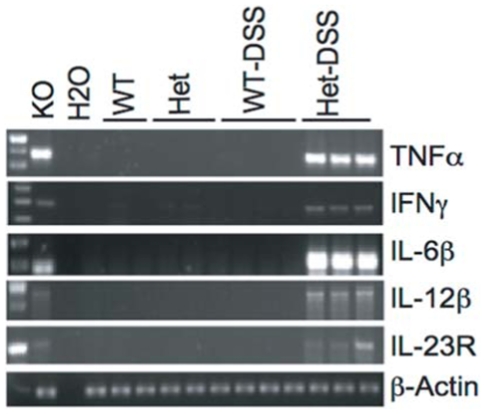
Colonic up-regulation of cytokines mRNA expression. DSS-treated TC-PTP^+/−^(Het-DSS) mice showed up-regulation of TNFα, IFNγ, IL-12 β, IL-23R and IL-6. Untreated TC-PTP^−/−^ (null) mouse colon mRNA was used as a control.

Since there was hyper-activation of several STATs and JAKs mediators, we measured the expression of several corresponding downstream pro-inflammatory cytokines using RT-PCR. Colonic extracts from heterozygous TC-PTP mice treated with DSS showed up-regulation of TNFα, IFNγ, IL-12 β, IL-23R and IL-6 (Figure 5). Levels of these cytokines were significantly higher in DSS-treated TC-PTP^+/−^ mice and untreated TC-PTP^−/−^ (KO, used as a control) mice compared to WT mice, TC-PTP^+/−^ (DSS non treated) and WT (DSS-treated).

**Figure 6:**
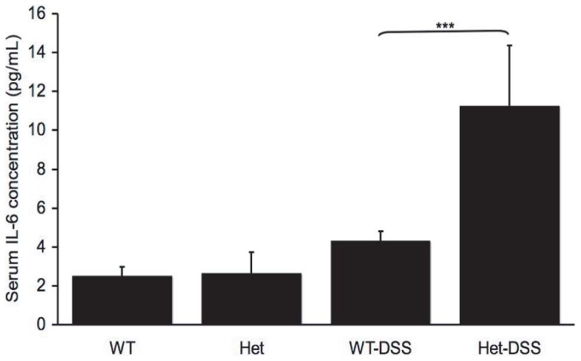
IL-6 serum level in DSS-treated and control mice. Serum was collected at the time of euthanasia and analyzed for cytokine levels using Mouse Cytokine Multiplex Assay. A 4.2-fold increase in serum IL-6 concentration in the TC-PTP^+/−^(Het-DSS) (11.6±7.6 pg/ml) compared to TC-PTP^+/+^ (WT-DSS) (2.6±2.4 pg/ml) mice (p<0.05).

Based on published evidence that Jaks and STAT pathways negatively regulate inflammatory bowel diseases like colitis in Crohn's disease and ulcerative coloitis [1]–[7], we pursued the possibility that DSS treatment was also initiating systemic inflammation in TC-PTP^+/−^ mice. Therefore, we measured the levels of IL-6, IL-12p40, IFN-γ and TNF-α in the serum of DSS-treated TC-PTP^ +/−^ and TC-PTP^+/+^ mice as well as non-treated control animals. Of the cytokines analyzed, we were able to detect a 4.2-fold increase in serum IL-6 concentration in the TC-PTP^+/−^ mice treated with DSS compared to TC-PTP^+/+^ mice treated with DSS (*p*<0.05) (Figure 6).

## Discussion

Much effort has recently been invested in identifying new associations between gene expression and complex disease traits such as in IBD. Recently, the Wellcome Trust Case Control Consortium and other groups identified SNPs associated with IBD in close proximity to the TC-PTP locus [1]–[7]. Given these results, and the systemic inflammatory phenotype identified in TC-PTP^−/−^ mice [8], we used a well established model of experimental colitis to study the role of TC-PTP in bowel inflammation. Since the homozygous TC-PTP^−/−^ phenotype is lethal at approximately 3 weeks of age, we focused our efforts on the hemizygous TC-PTP knock-out animals [8]. The gene dosage reduction of TC-PTP enzyme in heterozygous mice was associated with an increased susceptibility to DSS-induced colitis. DSS-treated TC-PTP^+/−^ mice lost more body and bowel weight than control animals (Figure 1). They showed more pronounced decrease in number, height and width of colonic crypts (Figure 2), and higher histo-pathological scoring of colonic inflammatory changes (Figure 3). Also, DSS-treatment in TC-PTP^+/−^ mice was found to result in colonic hyper-phosphorylation of the transduction pathway mediators JAK1, JAK3, STAT1, STAT3 and STAT5, and further decrease in TC-PTP protein levels (Figure 4), as well as up-regulation of mRNA transcripts for TNFα, IFNγ, IL-12 β, IL-23R and IL-6 (Figure 5). Systemically, we detected a significant increase in peripheral blood lymphocyte and neutrophil counts (Figure 3) and a significant increase in serum IL-6 concentration in the DSS-treated TC-PTP^+/−^ mice.

Taken together, these novel results indicate that DSS colitis inflammation is more severe in TC-PTP ^+/−^ mice than in control animals. This is accompanied by a marked increase in local expression of TC-PTP regulated inflammation mediators and a concomitant further decrease in TC-PTP expression. This local inflammation is associated with a systemic inflammatory response characterized by an elevation in serum IL-6 levels and increased peripheral blood. Thus, not only TC-PTP^+/−^ mice are more sensitive to DSS-induced colitis, but they also respond more severely to DSS-treatment both at the local and systemic levels.

Noteworthy, the overexpression of mRNA transcripts of IFN-γ, TNF-α and IL-12, and the systemic increase in IL-6 serum levels observed in DSS-treated TC-PTP^+/−^ mice are similar to those observed in control TC-PTP^−/−^ mice. The over-expression of T_H1_ cytokines has previously been associated with Crohn's disease [8] [17]. Increased levels of those pro-inflammatory cytokines are a major factor in organ-specific autoimmune inflammation [3], [7]. Looking at events downstream from cytokine production, analysis of the status of signal transducers revealed increased phosphorylation of Jak1 and Jak3 and of their substrates STAT1, STAT3 and STAT5 in DSS-treated TC-PTP^+/−^ mice in a manner reminiscent to what is observed in TC-PTP^−/−^ mice [8].

In the DSS-induced colitis mouse model, initial injury leads to its disruption and to the entry of bacteria into the intestinal wall and into the blood stream. Supporting evidence for this comes from rats treated with DSS which demonstrated higher level of endotoxin in the portal blood than control animals [18]. Bacterial invasion of the colonic wall and consequent production of endotoxins such as LPS in turn cause the recruitment and activation of inflammatory cells and result in the upregulation of multiple inflammatory cytokines (Figure 5) [18] [17], [19]. One phenomenon occurring in DSS-treated TC-PTP^+/−^ mice as well as in patients with Crohn's disease is the over-production of IL-6 [20] [21] [22]. Indeed, levels of IL-6 correlate with disease severity in humans [23]. Binding of IL-6 to its receptor initiates cellular events, including activation of Jak kinases. Jaks are a class of tyrosine kinases that associate with cytokine receptors and are able to phosphorylate and activate STAT proteins, which then dimerize and enter the nucleus to activate the transcription of target genes [24]. Jaks 1 and 3 have been identified as substrates of TC-PTP downstream of IL-2 signaling in substrate-trapping experiments using hematopoietic cell lines [25]. Downstream of Jak activation, STATs are phosphorylated and can thus be modified by PTPs to downregulate their action [26]. For instance, IL-6-induced activation of STAT3 was suppressed by over expression of the nuclear isoform of TC-PTP [27]. Thus, TC-PTP function has an important impact on the sensitivity of the innate immune system to bacterial antigens such as LPS [8], TC-PTP^−/−^ macrophages are hypersensitive to LPS and respond by producing exaggerated amounts of interferon-alpha (IFN-α), tumor necrosis factor-gamma (TNF-γ), interleukin-12 (IL-12), and nitric oxide (NO) [28]. Likewise, cells lacking TC-PTP produced increased levels of IL-6 upon TNF-alpha (TNF-α) treatment [29]. TC-PTP^−/−^ bone marrow-derived macrophages have elevated levels of phosphorylated Jak 1 upon IFN-gamma (IFN-γ) stimulation [25].

Here we show that a gene dosage reduction of TC-PTP enzyme in heterozygous TC-PTP^+/−^ mice directly affects the intensity of the inflammatory response to colonic mucosal injury. This is mediated by an increased production of cytokines, including IL-6, which in all likelihood results directly from the hemi-deficiency in TC-PTP. Increased cytokine levels result in increased phosphorylation of Jaks tyrosine kinases, which in turn phosphorylate/activate signal transducers and activators of transcription (STATs). Owing to the hemi-deficiency of TC-PTP in TC-PTP^+/−^ mice, normal targets for TC-PTP dephosphorylation, i.e. Jak-1, Jak3, and STAT1, STAT3 and STAT5 [9] , remain aberrantly activated, leading to an exaggerated inflammatory response. Our novel findings open the way for future studies examining the unexpected decrease in TC-PTP^+/−^ protein levels following DSS treatment, as well as identifying specific mechanisms leading to the loss of epithelial cells in this animal model of IBD. Furthermore we will be able to investigate whether epithelial cell death is secondary to the decreased expression of TC-PTP in hemi-deficiency using isolated colonic epithelial cells and in particular, mucosal immune cells (lamina propria mononuclear cells/LPMC or lamin propria Tcells/LPT). By inference, human beings with a constitutively less active or less abundant TC-PTP enzyme might be more susceptible to exaggerated bowel inflammation in case of bowel mucosal injury. This hypothesis is consistent with WTCC's observation of Crohn's disease being associated with SNPs mapping near the TC-PTP gene locus.

## Materials and Methods

### Animal Model

#### Mice

Experiments were performed with Balb/c mice [10]. TC-PTP^+/+^ and TC-PTP^+/−^mice were obtained from heterozygous mating and genotyped by Southern blotting as previously described [10]. All mice were kept in specific pathogen-free housing in the animal care facility.

### Ethics Statement

All procedures were reviewed and approved from the McGill University Animal Care Ethics committee.

### Induction of Colitis

TC-PTP ^+/+^ and TC-PTP ^+/−^ mice, 19 days old and 11–12g in body weight, were administered 5% dextran sulfate sodium (DSS) dissolved in filter-purified and sterilized drinking *ad libitum* for 10 days, after which hydration with regular water was resumed for 1 day before they were weighted and humanely sacrificed (approx. age 30 days) [15], [16]. Animals were weighed daily and monitored for rectal bleeding, diarrhea, and general signs of morbidity. Control animals were given normal drinking water.

### Histology

#### Evaluation of inflammation activity

The large intestine was excised, and the colon weight was measured. The distal (2.2∼2.4 cm) of the colon was transected, flushed with PBS and areas of redness for gross inflammation were recorded. The macroscopic appearance of the colon was scored in a blinded fashion as follows: 0, no evidence of inflammation; 1, erythema only; 2, erythema and small erosions; 3, 2 or more bleeding ulcers and/or inflammation and/or moderate adhesions [30]. Tissues were then fixed with 4% formaldehyde overnight. After overnight dehydration and paraffin impregnation, tissues were embedded in a paraffin block and serially sectioned. The 5-µm-thick sections were cut and stained with hematoxylin and eosin (H & E) .The sections were microscopically examined for histopathological changes, inflammation severity was scored by a blinded pathologist.

### Blood Count

Blood samples were taken at the end of the experiments by cardiac puncture (heart blood) for blood count using an automatic cytometer following manufacturer protocol (Beckmann Coulter, Germany).

### Histological Scoring

Analysis was performed blinded by pathologist and each specimen was scored on a scale from 0 to 40 as follows: severity of inflammation (0–3: none, slight, moderate, severe), epithelial erosion (0–3: none, none, slight, moderate, severe), and crypt damage (0–4: none, basal 1/3 damaged, basal 2/3 damaged, only surface epithelium intact, entire crypt and epithelium lost). Each score was then multiplied by a factor equivalent with the percentage of tissue involvement (×1: 0–25%, ×2: 26–50%, ×3: 51–75%, ×4: 76–100%) [31] [32].

### Immunoblotting

Large bowel lysate was collected after crushing tissues in liquid nitrogen. Protein samples were resolved on 10% sodium dodecyl sulfate-polyacrylamide gel electrophoresis (SDS-PAGE) and subjected to immunoblotting. TC-PTP was detected using a monoclonal antibody 3E2 as described in [33]. Phosphorylated Jak1, Jak2, Jak3, Stat1, Stat3 and Stat5 proteins were detected using polyclonal antibodies (Cell Signaling Technology). Total Jak1, Jak2, Jak3, Stat1, Stat3 and Stat5 proteins were detected using polyclonal antibodies (Cell Signaling Technology). Calnexin and β-Actin monoclonal antibody were used as loading control for protein samples (Sigma, Oakville, ON, Canada). Primary antibodies were followed by horseradish peroxidase-conjugated goat anti–rabbit or mouse IgG (Perkin Elmer, Boston, MA) as appropriate. Proteins were detected by enhanced chemiluminescent substrate (Perkin Elmer, Boston, MA). Mean intensity values were calculated using ImageJ software (http://rsbweb.nih.gov/ij/).

### RT-PCR of mRNA

Total RNA was extracted from tissues using TRIzol (Invitrogen) according to the manufacturer's specifications. mRNA was transcribed using the Super ScriptIII (Invitrogen) reverse transcriptase-polymerase chain reaction (RT-PCR) kit (Qiagen, Mississauga, ON, Canada) and the PCR machine (Roche Diagnostics, Laval, QC, Canada).

### Primers

Oligo-nucleotides were synthesized by Sigma (Sigma, Oakville, ON, Canada) and sequences are as follows:

β-Actin was used as control of 187bp as forward, 5′-AGGTGACAGCATTGCTTCTG-3′ and reverse, 5′-GCTGCCTCAACACCTCAAC-3′. Interleukin-6 (IL-6) forward, 5′- TCCAGTTGCCTTCTTGGGAC -3′ and reverse, 5′- GTGTAATTAAGCCTCCGACTTG-3′ with size of fragment as 140 bp. Tumor necrosis factor-alpha (TNF-α) forward 5′- CCTGTAGCCCACGTCGTAGC -3′ and reverse, 5′- TTGACCTCAGCGCTGAGTTG -3′ with size of fragment as 373 bp. Interferon-gamma (IFN-γ) forward 5′-TGGAGGAACTGGCAAAAGGATG -3′, and reverse 5′-CGCTTCCTGAGGCTGGATTC -3′, with size of fragment as 289 bp. Interleukin-12β-p40 (IL-12β-p40) forward 5′-AGATGACATCACCTGGACCTCAG -3′, and reverse 5′- ACGTGAACCGTCCGGAGTAA -3′, with size of fragment as 230 bp.

Interleukin 23 receptor (IL-23R) forward, 5′-TCCACTGACTCACTGCAAGG -3′ and reverse 5′-GTTCGTGGGATGATTTTGCT -3′, with size of fragment as 500 bp.

### Serum Cytokine Analysis

Serum levels for IL-6, TNF-α, IFN-γ, and IL-12p40 were measured using Bio-Plex Pro Mouse Cytokine Multiplex Assay (Bio-Rad Laboratories, Hercules, CA, USA) as per manufacturer's protocol with some minor modifications. In brief, 25 µl of pre-mixed beads coated with capture antibodies were added to a 96-well plate. Beads were washed twice and 25 µl of standards and serum (diluted 4-fold) were added to the wells before incubating on a microtiter plate shaker at 300 rpm, protected from light, for one hour at room temperature. Beads were washed three times, followed by the addition of 12.5 µl of detection antibodies. The plate was incubated as before. After three washes, 25 µl of streptavidin-phycoerythrin were added to each well and incubated as before for only 10 minutes. Beads were resuspended in 62.5 µl Bio-Plex Assay buffer on the shaker at 300 rpm. Beads were read using the Bio-Plex Reader (Bio-Rad Laboratories, Hercules, CA) and data was analyzed with Bio-Plex Manager 2.0 software (Bio-Rad Laboratories, Hercules, CA).

### Statistical Analysis

A linear-mixed model was employed and log likelihood was used to test significance between genotypes by treatment over time using R Statistical Package (R Development Core Team, http://www.r-project.org/).

Histograms represent mean ± standard error of the mean (SEM). *P* values were determined using a one-way ANOVA using Tukey-Kramer Multiple Comparisons.
